# Phenyl-Containing Silicone–Acrylate Core–Shell Particles for Transparent Toughening of PMMA with Enhanced Property Retention Under UVA Aging

**DOI:** 10.3390/polym18202564

**Published:** 2026-10-17

**Authors:** Yuao Li, Fan Yang, Xiang Liu, Runxiong Lin, Tao Zhuang

**Affiliations:** 1 School of Polymer Science and Engineering, Qingdao University of Science and Technology, Qingdao 266042, China; qdlya@mails.qust.edu.cn (Y.L.); xiangliu2001l@mails.qust.edu.cn (X.L.); 2 School of Materials Science and Engineering, Ocean University of China, Qingdao 266404, China; fanyang@stu.ouc.edu.cn

**Keywords:** poly(methyl methacrylate), silicone–acrylate core–shell particles, transparent toughening, refractive-index contrast, UVA aging performance

## Abstract

Transparent toughening of poly(methyl methacrylate) (PMMA) requires simultaneous control of mechanical energy dissipation and optical scattering, together with retention of properties under ultraviolet exposure. Here, silicone–acrylate (Si–ACR) core–shell particles comprising a PHSO-derived silicone-rich core and a PMMA-rich outer region were prepared by two-stage seeded emulsion polymerization without a deliberately added grafting agent. The phenyl-containing silicone-rich phase was selected to reduce the anticipated refractive-index contrast with PMMA. Si–ACR particles with an average diameter of 275 nm provided a favorable mechanical–optical balance. At 15 wt% Si–ACR, the notched Izod impact strength increased from 1.8 to 18.1 kJ/m^2^, while total luminous transmittance remained 91.4% and 90.8% for 1 and 2 mm specimens, respectively, with haze below 0.8%. Fracture morphology was consistent with local debonding and/or void formation followed by enhanced plastic deformation of the PMMA matrix. After 500 h of accelerated UVA exposure, the blend retained an impact strength of 16.8 kJ/m^2^ and a transmittance of 89.7% for the 1 mm specimen. These findings suggest potential applications in transparent protective shields and optical covers requiring improved impact resistance, low haze, and property retention under UVA exposure.

## 1. Introduction

Poly(methyl methacrylate) (PMMA) is widely used in transparent structural and optical components because of its high visible-light transmittance, dimensional stability, low density, and processability. Its intrinsic brittleness and notch sensitivity, however, limit applications requiring resistance to sudden impact. Incorporating a dispersed elastomeric phase is an established route to increase energy absorption, and core–shell impact modifiers are particularly useful because particle architecture, dimensions, shell chemistry, and interfacial interactions can be independently adjusted. Recent studies on PMMA further demonstrate that modifier morphology and particle size strongly influence the attainable balance between impact toughness, stiffness, and transparency [1,2,3].

For transparent PMMA, the challenge is not simply to increase impact strength but also to limit the optical penalty associated with a dispersed toughening phase. Light scattering depends on the refractive-index (RI) contrast between phases, particle dimensions, aggregation, and phase morphology, whereas mechanical energy dissipation also depends on the deformability of the dispersed phase and its interactions with the matrix. Refractive-index-controlled core–shell particles and related transparent multiphase systems have demonstrated approaches to mitigating optical scattering [4,5,6,7]. Nevertheless, the particle characteristics favorable for impact toughening do not necessarily coincide with those that maximize optical clarity. Therefore, particle architecture, dimensions, dispersion, and matrix compatibility must be considered concurrently when designing transparent impact-modified PMMA.

Silicone-containing elastomers offer an attractive compliant phase for impact modification, while incorporation of phenyl groups can increase the RI of polysiloxanes, providing a potential means of reducing optical contrast with PMMA [8]. Silicone–acrylate core–shell modifiers have been investigated for improving the toughness of PVC, and silicone-containing multilayer modifiers have also been explored in ASA systems [9,10]. Separately, previous studies have documented changes in the mechanical and optical properties of PMMA under ultraviolet-related aging conditions [11,12]. Although these investigations provide important foundations for modifier design and durability assessment, they do not fully establish how a phenyl-containing silicone-rich core surrounded by a PMMA-rich outer region performs in terms of impact toughness, optical transparency, and property retention after UVA exposure. In particular, the combined effects of particle dimensions and modifier content on these properties remain insufficiently characterized for this specific architecture. Addressing this relationship is relevant to transparent protective and optical components requiring both impact resistance and optical clarity during environmental exposure.

**Figure 1:**
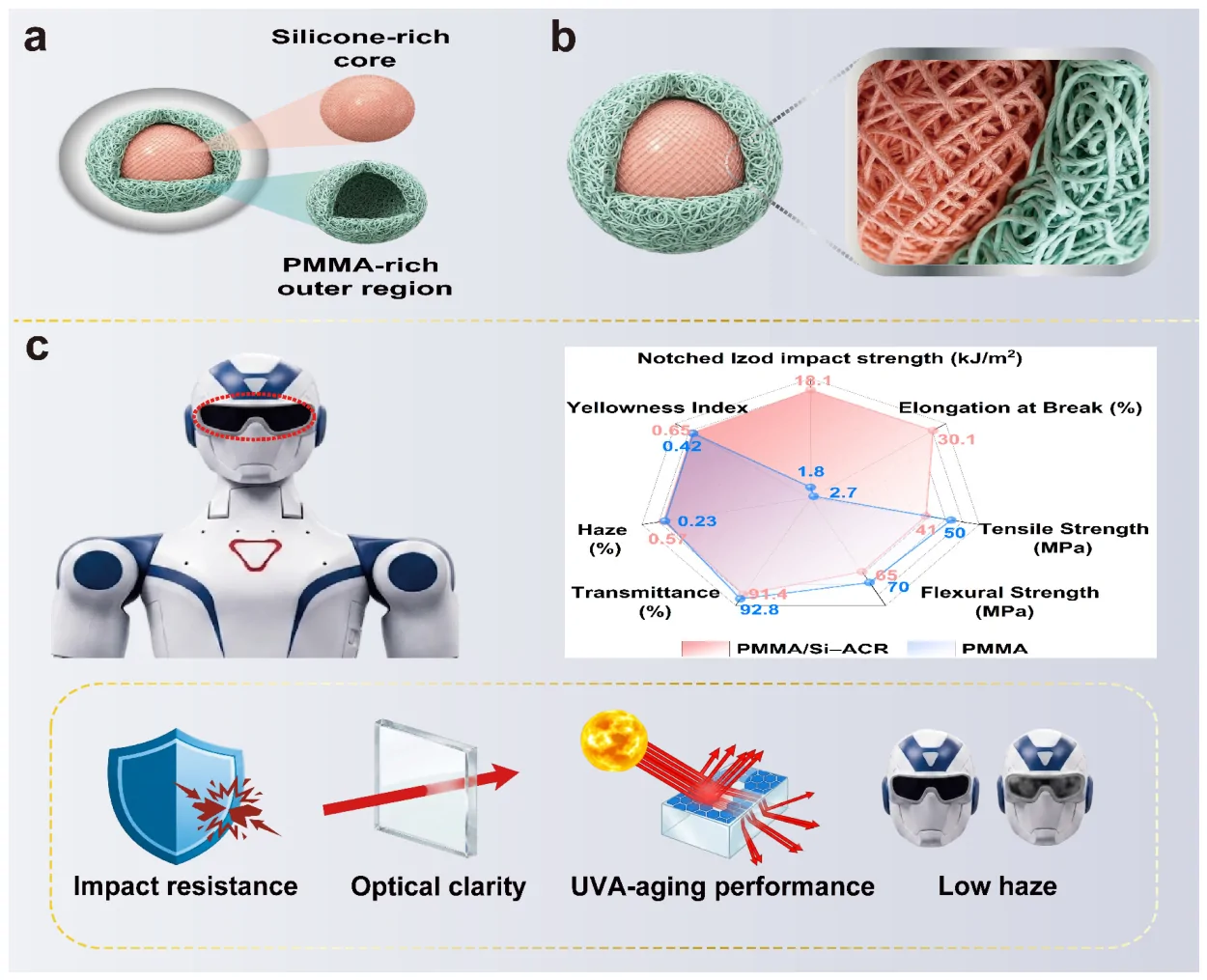
Design concept and potential applications of phenyl-containing silicone–acrylate (Si–ACR) core–shell modifiers for transparent toughening of PMMA. (**a**) Schematic illustration of a Si–ACR particle comprising a phenyl hydrogen silicone oil (PHSO)-derived silicone-rich core and a PMMA-rich outer region. (**b**) Proposed interfacial configuration between the silicone-rich core and PMMA-rich outer region, without implying a specific chemical bonding mechanism. (**c**) Conceptual illustration of potential applications in transparent protective components, together with a comparison of selected mechanical and optical properties of the PMMA control and PMMA/Si–ACR blend and schematic representations of impact resistance, optical clarity, low haze, and property retention following accelerated UVA exposure. The yellow dashed box in (**c**) highlights schematic illustrations of impact resistance, optical clarity, UVA-aging performance, and low haze.

In this work, two-layer silicone–acrylate (Si–ACR) core–shell particles comprising a phenyl hydrogen silicone oil (PHSO)-derived silicone-rich core and a PMMA-rich outer region were prepared by two-stage seeded emulsion polymerization (Figure 1a). The phenyl-containing silicone-rich phase was selected to provide a compliant domain while potentially reducing the RI contrast with PMMA, whereas the PMMA-rich outer region was intended to favor compatibility with the surrounding matrix. No dedicated silane coupling agent or grafting crosslinker was intentionally introduced during formation of the outer region, and the proposed interfacial configuration is illustrated in Figure 1b without assuming a specific chemical bonding mechanism. The effects of particle size and modifier content on mechanical properties, optical transparency, fracture morphology, and property retention during accelerated UVA exposure were systematically evaluated. The blends were prepared by melt compounding and injection molding, providing an initial assessment using processing methods relevant to thermoplastic manufacturing, although industrial-scale production and long-term outdoor performance remain to be established. Figure 1c illustrates the potential application context of the developed material in transparent impact-resistant protective components.

## 2. Materials and Methods

### 2.1. Materials

Poly(methyl methacrylate) (PMMA; ACRYREX^®^ CM-211; density = 1.19 g/cm^3^; melt flow rate (MFR) = 16 g/10 min at 230 °C/3.8 kg according to ASTM D1238-23 [13]) was supplied by Zhenjiang CHIMEI Chemical Co., Ltd. (Zhenjiang, China). Phenyl hydrogen silicone oil (PHSO; active hydrogen content ≈ 0.75 wt%) was purchased from Zhejiang Runhe Chemical New Material Co., Ltd. (Hangzhou, China). Divinylbenzene (DVB, 80%), methyl methacrylate (MMA, 99%), and octylphenol polyoxyethylene ether (OP-10) were obtained from Shanghai Macklin Biochemical Co., Ltd. (Shanghai, China). Dipotassium alkenylsuccinate (LATEMUL ASK, 28 wt% active matter) was supplied by Kao Corporation (Tokyo, Japan). Potassium persulfate (KPS, 99.5%), sodium bicarbonate (NaHCO_3_, analytical grade), and concentrated sulfuric acid (H_2_SO_4_, analytical grade) were supplied by Sinopharm Chemical Reagent Co., Ltd. (Shanghai, China). Ethylene bis(stearamide) (EBS) and antioxidant 1076 were supplied by Qingdao Sainuo Chemical Co., Ltd. (Qingdao, China). Before use, MMA was washed with 5 wt% aqueous NaOH to remove the inhibitor, rinsed with deionized water to neutral pH, dried over anhydrous MgSO_4_, and distilled under reduced pressure. Concentrated H_2_SO_4_ was diluted with deionized water before use to prepare the aqueous coagulating solution. All other reagents were used as received.

### 2.2. Preparation of Si–ACR Core–Shell Particles

Si–ACR core–shell particles were prepared by two-stage seeded emulsion polymerization. A series of Si–ACR particles with average hydrodynamic diameters of approximately 196, 235, 275, 290, and 319 nm was obtained by varying the dosages of OP-10 and LATEMUL ASK while maintaining their relative proportion, the PHSO-to-MMA feed ratio, and the other polymerization conditions unchanged. The detailed formulations for the five particle-size series are provided in Table S2 of the Supplementary Materials. In the first stage, PHSO and DVB were emulsified in an aqueous medium containing OP-10 and LATEMUL ASK, with NaHCO_3_ used as a buffer. After addition of an aqueous KPS solution, the reaction was conducted at 75 °C under nitrogen for 30 min to obtain a PHSO-derived silicone-rich seed latex. In the second stage, an MMA pre-emulsion containing OP-10, LATEMUL ASK, NaHCO_3_, and deionized water was fed into the seed latex together with an additional aqueous KPS solution at 75 °C over 120 min. After completion of the feed, the reaction mixture was aged at 75 °C for 60 min to increase monomer conversion. No dedicated silane coupling agent or grafting crosslinker was intentionally added during formation of the PMMA-rich outer region. The resulting latex was cooled to room temperature and coagulated using an aqueous sulfuric acid solution prepared in advance by diluting concentrated H_2_SO_4_ with deionized water. The coagulated product was filtered, thoroughly washed with deionized water, and vacuum-dried to obtain the Si–ACR powder.

### 2.3. Preparation of PMMA/Si–ACR Blends

PMMA and Si–ACR were melt blended using a co-rotating twin-screw extruder (SHJ-20, Nanjing Jieya Extrusion Equipment Co., Ltd., Nanjing, China) equipped with conveying elements and two kneading-block sections to facilitate distributive and dispersive mixing. The screw speed and feeding rate were maintained at 85 rpm and 2.0 kg/h, respectively. The barrel temperatures from the feeding zone to the die were set at 190, 210, 220, 230, and 225 °C, respectively. Si–ACR contents of 5, 10, 15, 20, and 25 wt% were investigated. Each formulation contained 0.5 wt% EBS and 0.5 wt% antioxidant 1076, with the PMMA content adjusted accordingly.

The extrudates were pelletized, dried, and subsequently injection molded into standard test specimens. The WELLTEC injection molding machine (TTI-100D, Donghua Machinery Ltd., Dongguan, China) was equipped with four independently controlled temperature zones, comprising the rear, middle, and front barrel zones and the nozzle. The corresponding temperatures were set at 200, 215, 230, and 230 °C, respectively, providing a gradual temperature increase along the barrel. The injection pressure and mold temperature were maintained at 80 MPa and 70 °C, respectively, with a mold cooling time of 30 s. The same processing conditions were applied to all formulations to minimize processing-related variations and facilitate comparison of the effects of Si–ACR content. Detailed blend compositions are provided in Table S3 of the Supplementary Materials.

### 2.4. Characterization

For structural and morphological characterization, the PHSO-derived silicone-rich seed and the final Si–ACR particles corresponding to the formulation with a final average hydrodynamic diameter of approximately 275 nm were used unless otherwise specified. For mechanical and optical testing, at least five replicate specimens were evaluated for each condition. Mean values are reported in the tables, and error bars in the corresponding figures represent standard deviations.

Particle size analysis. The average hydrodynamic diameters of the synthesized latexes were determined by dynamic light scattering (DLS) using a Zetasizer Nano ZS90 particle-size analyzer (Malvern Panalytical, Malvern, UK) equipped with a 4 mW He–Ne laser operating at 633 nm and a 90° scattering configuration. The measurements were performed at 25 °C after appropriate dilution of the latexes with deionized water. For each formulation, three independently diluted aliquots from the same latex batch were prepared, with three consecutive measurements performed for each aliquot. The Z-average hydrodynamic diameter and polydispersity index (PDI) were obtained from cumulants analysis of the intensity autocorrelation function, while the particle-size distributions were evaluated using intensity-weighted distribution analysis. The results were expressed as mean ± standard deviation (SD), calculated from the three independently prepared aliquots (*n* = 3), with replicate measurements averaged for each aliquot. The corresponding particle-size distribution characteristics and PDI values are summarized in Table S6 of the Supplementary Materials.

Transmission electron microscopy (TEM). The morphology of the PHSO-derived silicone-rich seed particles and the final Si–ACR core–shell particles was examined using a Tecnai F20 transmission electron microscope (FEI, Hillsboro, OR, USA) operated at an accelerating voltage of 200 kV. Diluted latex samples were deposited onto carbon-coated copper grids and negatively stained with phosphotungstic acid for 3 min before observation to enhance phase contrast.

Scanning electron microscopy (SEM). Impact-fracture surfaces were examined using a JSM-6510 scanning electron microscope (JEOL, Tokyo, Japan). Before observation, the fracture surfaces were sputter-coated with a thin gold layer using a JFC-1600 auto fine coater (JEOL, Tokyo, Japan) to minimize surface charging.

X-ray diffraction (XRD). XRD patterns were recorded using a D/MAX-2500PC diffractometer (Rigaku Corporation, Tokyo, Japan) with Cu Kα radiation (λ = 1.5406 Å) operated at 40 kV and 100 mA. The diffraction patterns were collected over a 2θ range of 5–80° at a scanning rate of 3°/min.

Fourier transform infrared (FTIR) spectroscopy. FTIR spectra of the PHSO-derived silicone-rich seed material and Si–ACR particles were recorded using a Nicolet iS10 spectrometer (Thermo Fisher Scientific, Waltham, MA, USA). Samples were prepared as KBr pellets, and spectra were collected over the range of 400–4000 cm^−1^ at a resolution of 4 cm^−1^ with 32 accumulated scans.

Raman spectroscopy. Raman spectra of the PHSO-derived silicone-rich seed material and Si–ACR particles were recorded using an inVia Qontor confocal Raman microscope (Renishaw, Wotton-under-Edge, UK) equipped with a 633 nm excitation laser and a 1200 lines/mm grating. Spectra were collected over the range of 200–4000 cm^−1^ at room temperature.

X-ray photoelectron spectroscopy (XPS). Surface chemical compositions of the PHSO-derived silicone-rich seed material and Si–ACR particles were analyzed using a Thermo Scientific K-Alpha X-ray photoelectron spectrometer (Thermo Fisher Scientific, Waltham, MA, USA) equipped with a monochromated Al Kα X-ray source (hν = 1486.6 eV). An X-ray spot size of 400 μm was used. Survey spectra were acquired at a pass energy of 200 eV with an energy step of 1.0 eV, whereas high-resolution C 1s and Si 2p spectra were collected at a pass energy of 50 eV with an energy step of 0.1 eV. Binding energies were referenced to the adventitious C 1s signal at 284.8 eV, and the high-resolution spectra were deconvoluted using Avantage software (version 5.99).

Mechanical properties. Notched Izod and Charpy impact strengths were measured using a GT-7045-MD pendulum impact tester (Gotech Testing Machines (Dongguan) Co., Ltd., Dongguan, China) with 2.75 and 2 J pendulums, respectively, according to ISO 180:2023 (specimen type 1A) [14] and ISO 179-1:2026 (test configuration 1eA) [15]. Tensile strength and elongation at break were measured using a GT-TCS-200 universal testing machine (Gotech Testing Machines (Dongguan) Co., Ltd., Dongguan, China) at a crosshead speed of 50 mm/min according to ISO 527-2:2025 [16]. Tensile elastic modulus was determined in separate tests at a crosshead speed of 1 mm/min using a calibrated extensometer, with the modulus calculated by linear regression over the strain interval of 0.05–0.25% in accordance with ISO 527-1:2019 [17]. Representative engineering stress–strain curves were obtained from the recorded tensile-test data. Flexural properties were measured using a GT-TCS-2000 testing machine (Gotech Testing Machines (Dongguan) Co., Ltd., Dongguan, China) at a crosshead speed of 2 mm/min according to ISO 178:2019 [18].

Optical properties and accelerated UVA aging. PMMA/Si–ACR blends were injection molded into plates with thicknesses of 1 and 2 mm. Total luminous transmittance was measured using a WGT-S transmittance/haze meter (Shanghai INESA Physico-Optical Instrument Co., Ltd., Shanghai, China) according to ISO 13468-1:2019 [19], and haze was measured using the same instrument according to ISO 14782:2021 [20]. Yellowness index (YI) was determined using an Agera L2 spectrophotometer (HunterLab, Reston, VA, USA) according to ISO 17223:2026 [21]. Accelerated UVA aging was performed using a QUV/se accelerated weathering tester (Q-Lab Corporation, Westlake, OH, USA) equipped with UVA-340 fluorescent lamps according to ISO 4892-3:2024 [22]. Each cycle consisted of 8 h of UV irradiation at a black-panel temperature of 60 °C and an irradiance of 0.76 W/(m^2^·nm) at 340 nm, followed by 4 h of condensation at 50 °C. Specimens were evaluated after 240 and 500 h of exposure. After each exposure period, optical transmittance and notched Izod impact strength were measured to determine property retention.

Melt flow rate. The melt flow rate (MFR) of the PMMA/Si–ACR blends was measured using a GT-7100-MI melt-flow tester (Gotech Testing Machines (Dongguan) Co., Ltd., Dongguan, China) at 230 °C under a load of 10 kg according to ISO 1133-1:2022 [23].

### 2.5. Preparation of Illustrative Graphics

Generative artificial intelligence was used solely to create limited non-data-bearing illustrative elements in Figure 1c and the graphical abstract for visualization of the potential application context of the material. These illustrative elements were generated using Nano Banana Pro (Gemini 3 Pro Image, Google) and were subsequently reviewed, selected, edited, and incorporated into the final composite graphics by the authors using Adobe Illustrator 2026. No generative AI was used to generate, modify, analyze, or interpret any experimental data, microscopy images, spectra, quantitative plots, molecular structures, or numerical results presented in this study. The core–shell particle renderings were created using Cinema 4D R26, the three-dimensional molecular structures were prepared using ChemDraw 3D 2023, experimental data plots were generated using Origin 2021, and the final figure layouts were assembled using Adobe Illustrator 2026.

## 3. Results and Discussion

### 3.1. Structural Characterization and Morphological Evolution of Si–ACR Core–Shell Particles

**Figure 2:**
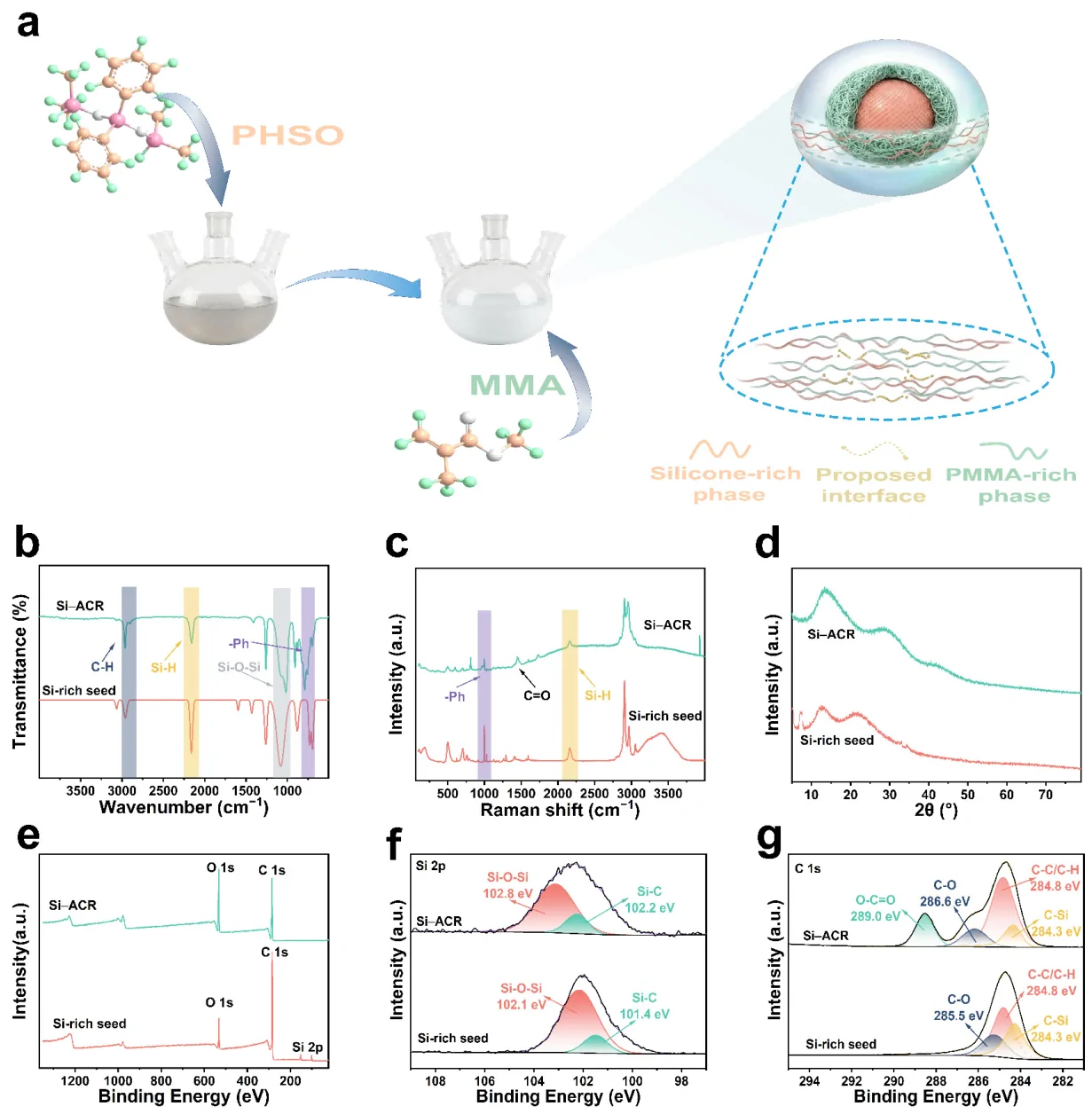
Structural and surface-composition characterization of the PHSO-derived silicone-rich seed and Si–ACR particles corresponding to the formulation with a final average hydrodynamic diameter of approximately 275 nm. (**a**) Schematic illustration of the two-stage seeded emulsion polymerization process. The arrows in (**a**) indicate the sequential formation of the PHSO-derived silicone-rich seed and subsequent MMA polymerization to form the PMMA-rich outer region. (**b**) FTIR spectra, (**c**) Raman spectra, and (**d**) XRD patterns of the PHSO-derived silicone-rich seed and Si–ACR particles. (**e**) XPS survey spectra and high-resolution (**f**) Si 2p and (**g**) C 1s spectra showing changes in near-surface composition after MMA polymerization.

**Figure 3:**
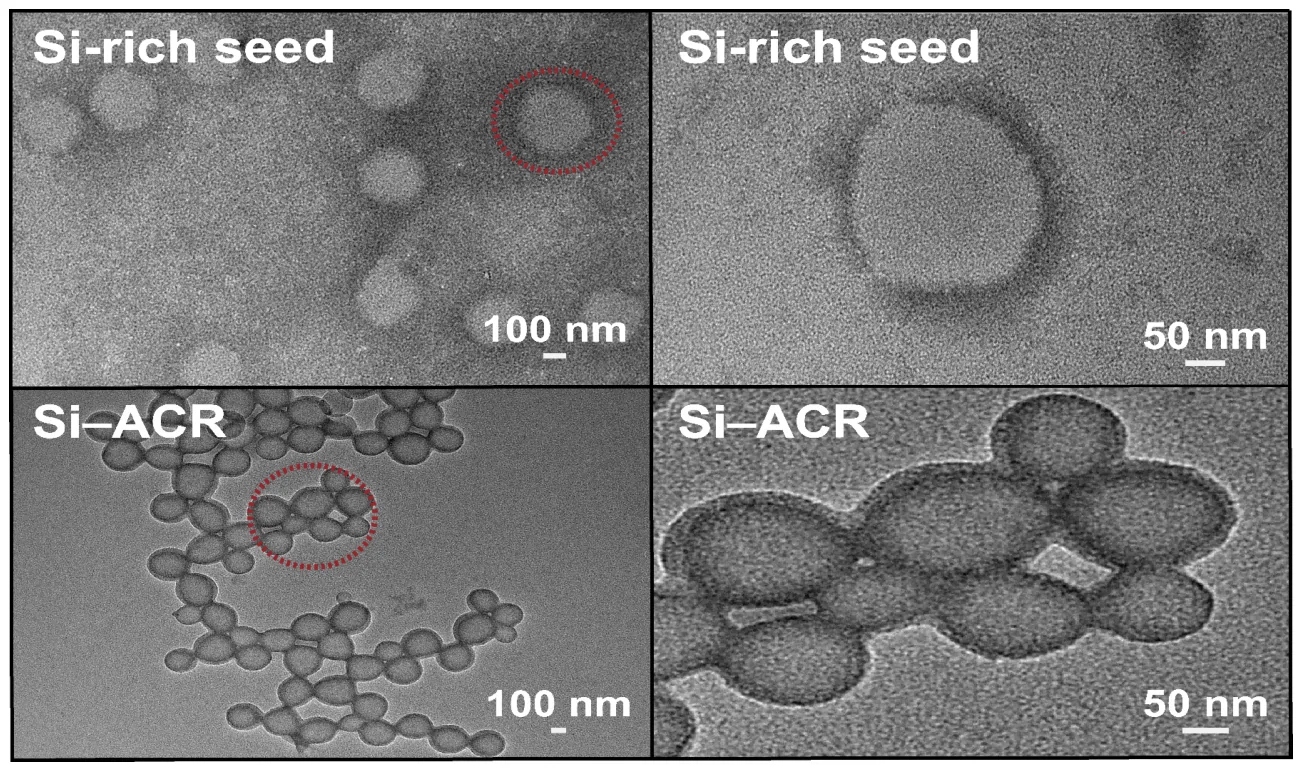
TEM images of the PHSO-derived silicone-rich seed (**upper row**) and final Si–ACR particles (**lower row**) corresponding to the formulation with a final average hydrodynamic diameter of approximately 275 nm. The left and right columns show images at different magnifications, with scale bars of 100 and 50 nm, respectively. The red dashed circles highlight representative particle regions in the lower-magnification images. The contrast variations observed in some final Si–ACR particles are consistent with a radially heterogeneous morphology.

The structural and morphological evolution from the PHSO-derived silicone-rich seed to the final Si–ACR particles is summarized in Figure 2 and Figure 3. The FTIR spectrum of the silicone-rich seed exhibits the characteristic absorption features of the siloxane phase, including Si–O–Si, Si–H, and phenyl-related vibrations. After MMA polymerization, the Si–ACR spectrum retains the characteristic bands of the silicone component while exhibiting a pronounced ester C=O absorption attributable to PMMA. The Raman spectra show corresponding changes, with characteristic features associated with both the silicone and acrylic components observed in the final particles. The XRD patterns of both samples are dominated by broad diffuse halos, and no new sharp diffraction peaks appear after MMA polymerization, indicating that the particles remain predominantly amorphous. Collectively, these spectroscopic results demonstrate the coexistence of silicone- and PMMA-related chemical components in the final Si–ACR particles but do not, by themselves, establish their spatial core–shell arrangement.

XPS was therefore employed to examine the near-surface chemical composition of the particles. Because conventional XPS probes only the near-surface region, changes in elemental and chemical-state contributions can provide information on the composition of nanoparticle coatings [24,25]. Relative to the silicone-rich seed, the Si–ACR survey spectrum shows pronounced attenuation of the Si signal together with increased contributions from C and O. The Si 2p signal is substantially attenuated after MMA polymerization, consistent with reduced exposure of the silicone-rich phase at the particle surface. Meanwhile, the C 1s spectrum of Si–ACR contains pronounced O–C=O and C–O components at approximately 289.0 and 286.6 eV, respectively, which are characteristic of PMMA. The attenuation of the surface Si signal together with the emergence of PMMA-associated carbon environments supports enrichment of PMMA in the outer region of the Si–ACR particles. Because XPS is surface-sensitive rather than a direct probe of interfacial bonding, these results are interpreted as evidence for a PMMA-rich outer region and are not used to establish the chemical nature of the internal interface.

TEM was further employed to examine the particle morphology (Figure 3). Both the PHSO-derived silicone-rich seed and final Si–ACR particles exhibited approximately spherical morphologies. Following MMA polymerization, contrast variations between the central and peripheral regions of the Si–ACR particles were observed, consistent with a radially heterogeneous structure. Together with the two-stage seeded emulsion polymerization process, the coexistence of silicone- and PMMA-related components identified by FTIR and Raman spectroscopy, and the PMMA-enriched surface composition indicated by XPS, these observations support the proposed silicone-rich-core/PMMA-rich-outer-region architecture [26,27]. However, the core–shell boundaries were not sufficiently resolved for reliable quantitative determination of the PMMA-rich outer-region thickness, and complete shell coverage cannot be established from the present images alone.

### 3.2. Mechanical Properties and Fracture Behavior of PMMA/Si–ACR Blends

The effect of Si–ACR particle size on the mechanical performance of the blends was first examined at a fixed modifier content of 15 wt% (Figure S1a–c and Table S4). As the average particle diameter increased from 196 to 275 nm, the notched Izod impact strength increased from 14.7 to 18.1 kJ/m^2^, followed by a decrease to 16.3 kJ/m^2^ at 319 nm. The Charpy impact strength exhibited a similar overall trend, although its maximum value of 16.9 kJ/m^2^ occurred at 290 nm, compared with 16.7 kJ/m^2^ at 275 nm. This nonmonotonic dependence suggests that an intermediate particle size favors impact toughening under the present testing conditions. Cho et al. [28] reported maximum impact strength at a particle diameter of approximately 0.25 μm in a PBA/PMMA core–shell-modified PMMA system, while also demonstrating the influence of fracture testing conditions. Previous studies have further shown that core–shell interphase characteristics affect the mechanical behavior of rubber-toughened PMMA [29], whereas particle dimensions and spatial distribution influence toughening in other core–shell-modified glassy polymers [30]. These findings suggest that particle size may affect local stress development and the activation of matrix plastic deformation, although the precise deformation pathways were not directly resolved in the present study. Notably, increasing the particle diameter from 275 to 290 nm reduced the total luminous transmittance from 91.4% to 89.3% for 1 mm specimens and from 90.8% to 88.4% for 2 mm specimens (Table S4). Therefore, approximately 275 nm was selected as the preferred particle size for the subsequent composition study, considering its highest measured Izod impact strength and favorable balance between impact resistance and optical transparency.

**Figure 4:**
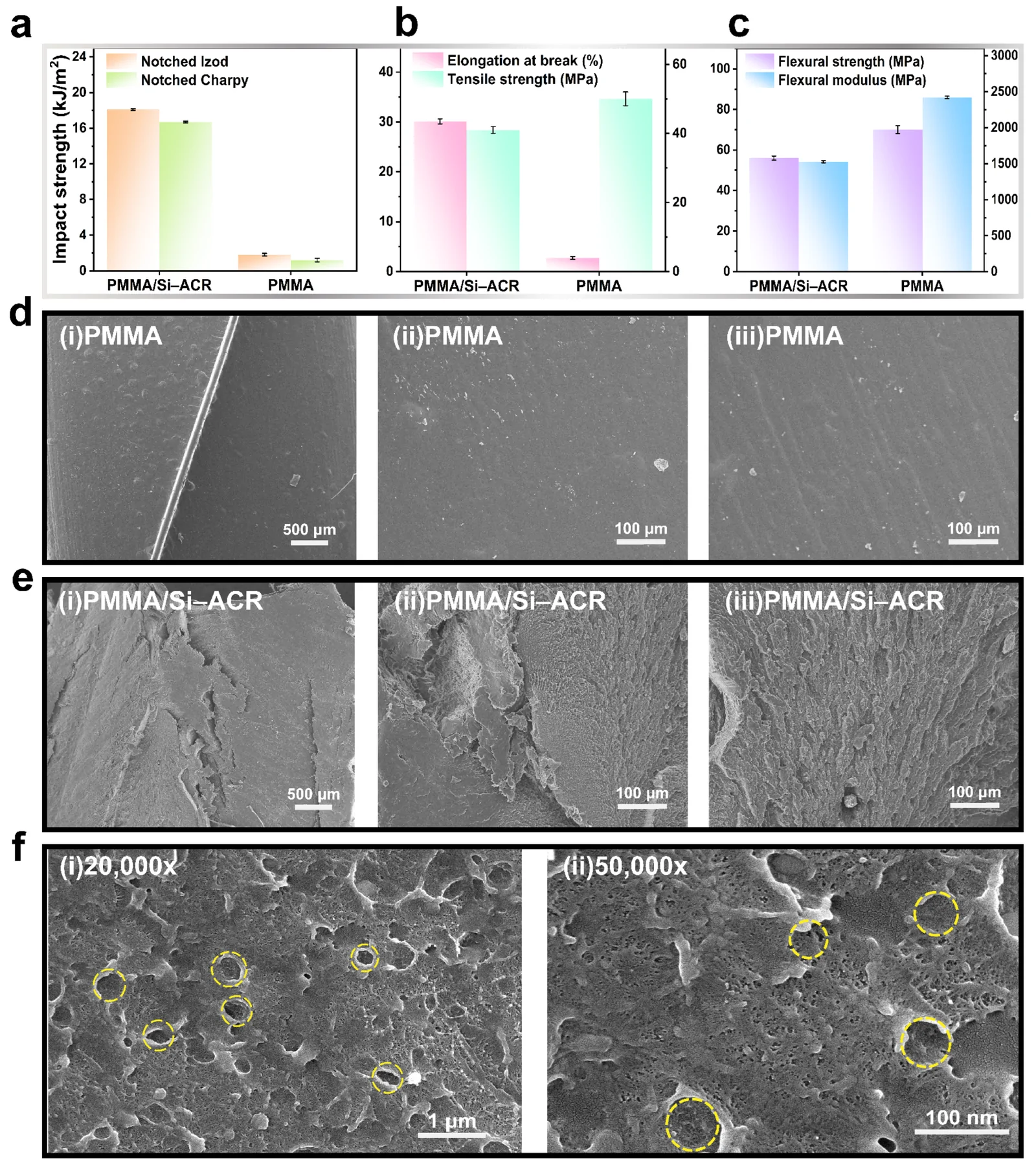
Mechanical properties and impact-fracture morphology of the PMMA control and PMMA/Si–ACR blend containing 15 wt% Si–ACR. (**a**) Notched Izod and Charpy impact strengths, (**b**) elongation at break and tensile strength, and (**c**) flexural strength and flexural modulus. SEM images of the impact-fracture surfaces of (**d**) the PMMA control and (**e**) the PMMA/Si–ACR blend at different magnifications. (**f**) Higher-magnification SEM images of the PMMA/Si–ACR fracture surface; the dashed circles indicate representative cavity-like features and apparent particle-pull-out sites. Error bars represent standard deviations from at least five replicate measurements (*n* ≥ 5).

With the average Si–ACR particle diameter fixed at approximately 275 nm, the effects of modifier content on the mechanical properties were then evaluated (Figure S1d–f and Table S5). The notched Izod impact strength increased from 1.8 kJ/m^2^ for the PMMA control to 10.3, 15.4, 18.1, and 19.8 kJ/m^2^ at Si–ACR contents of 5, 10, 15, and 20 wt%, respectively, before decreasing to 17.5 kJ/m^2^ at 25 wt%. At 15 wt% Si–ACR, the notched Charpy impact strength reached 16.7 kJ/m^2^, while the elongation at break increased from 2.7% for the PMMA control to 30.1% (Figure 4a,b). This improvement in ductility was accompanied by decreases in tensile strength from 50 to 41 MPa, flexural strength from 70 to 56 MPa, and flexural modulus from 2422 to 1526 MPa. Increasing the Si–ACR content from 15 to 20 wt% further improved the Izod impact strength by approximately 9.4%, but reduced the flexural modulus by 7.6%, from 1526 to 1410 MPa. Meanwhile, the total luminous transmittance decreased from 91.4% to 90.2% for 1 mm specimens and from 90.8% to 88.4% for 2 mm specimens, with the haze of the latter increasing from 0.73% to 0.79% (Table S5). Using a total luminous transmittance above 90% at both tested thicknesses as a comparative criterion, the 15 wt% formulation exhibited the highest Izod impact strength among the compositions satisfying this condition. Therefore, 15 wt% was selected as the preferred composition based on its overall balance of impact resistance, stiffness, and optical transparency, rather than the maximum impact strength alone. Similar toughness–stiffness trade-offs have been reported for core–shell-modified polymer systems [31,32,33].

The tensile deformation behavior was further examined using representative engineering stress–strain curves (Figure S3), with the corresponding tensile elastic moduli summarized in Tables S4 and S5. At a fixed Si–ACR content of 15 wt%, increasing the particle diameter from 196 to 319 nm reduced the tensile modulus from 2000 to 1800 MPa and the elongation at break from 35.0% to 27.5%, while the tensile strength remained within 39–43 MPa (Table S4). For the composition series using particles with an average diameter of approximately 275 nm, the tensile modulus decreased from 2500 MPa for the PMMA control to 1900 MPa at 15 wt% Si–ACR, corresponding to a reduction of 24.0%, and further decreased to 1730 MPa at 25 wt% Si–ACR (Table S5). The stress–strain curves show that the PMMA control fractured at a relatively low strain, whereas the Si–ACR-modified blends exhibited yielding followed by substantially extended tensile deformation (Figure S3). These results indicate that the incorporation of the compliant Si–ACR particles enhanced tensile deformability at the expense of axial stiffness, further illustrating the importance of balancing toughness and rigidity in the modified PMMA system.

The impact-fracture morphology provides further insight into the large difference between the PMMA control and the Si–ACR-modified material (Figure 4d–f). The PMMA control exhibits a comparatively smooth fracture surface with limited plastic deformation, consistent with brittle fracture. In contrast, the PMMA/Si–ACR blend shows a substantially rougher fracture surface containing numerous plastic ridges and deformation features. At higher magnification, localized cavity-like features and apparent particle-pull-out sites are visible in the modified material. Similar morphological features in rubber- and core–shell-toughened glassy polymers have been associated with local dilatational deformation and subsequent plastic deformation of the surrounding matrix [34,35]. Accordingly, the present SEM observations are consistent with local debonding, void formation, or a combination of both around the Si–ACR particles, followed by enhanced plastic deformation of PMMA. However, because these observations were obtained from post-fracture surfaces rather than TEM sections of the deformed blends, they do not establish the internal origin of the cavity-like features or distinguish silicone-core cavitation from core–shell interfacial separation, particle–matrix debonding, or particle pull-out. Accordingly, the mechanistic interpretation in Section 3.4 is presented as a plausible explanation consistent with the observed fracture morphology rather than as direct visualization of individual void-initiation processes.

### 3.3. Optical Properties and Accelerated UVA Aging Performance of PMMA/Si–ACR Blends

The effects of Si–ACR particle size and content on the optical properties of the blends are summarized in Figure S2 and Tables S4 and S5. At a fixed Si–ACR content of 15 wt%, increasing the particle diameter above approximately 275 nm resulted in a more pronounced decrease in transmittance. Increasing the Si–ACR content also gradually reduced transmittance and increased haze. Nevertheless, the blend containing 15 wt% Si–ACR with an average particle diameter of approximately 275 nm retained high optical clarity. Its total luminous transmittance was 91.4% and 90.8% for 1 and 2 mm specimens, respectively, compared with 92.6% and 92.8% for the PMMA control. The corresponding haze values were only 0.57% and 0.73%, and the yellowness indices were 0.68 and 0.65. These results indicate that substantial impact toughening was achieved with only a limited optical penalty. The phenyl-containing silicone-rich phase was selected to reduce the anticipated refractive-index contrast with PMMA. However, the approximate value of 1.49 adopted for optical-design estimation was not experimentally determined for the polymerized silicone-rich phase (Text S1 and Table S1 in the Supplementary Materials). Therefore, the observed transmittance and haze are consistent with the intended optical design but do not independently verify refractive-index matching, as particle dimensions, dispersion, and the PMMA-rich outer region also influence optical scattering [36].

**Figure 5:**
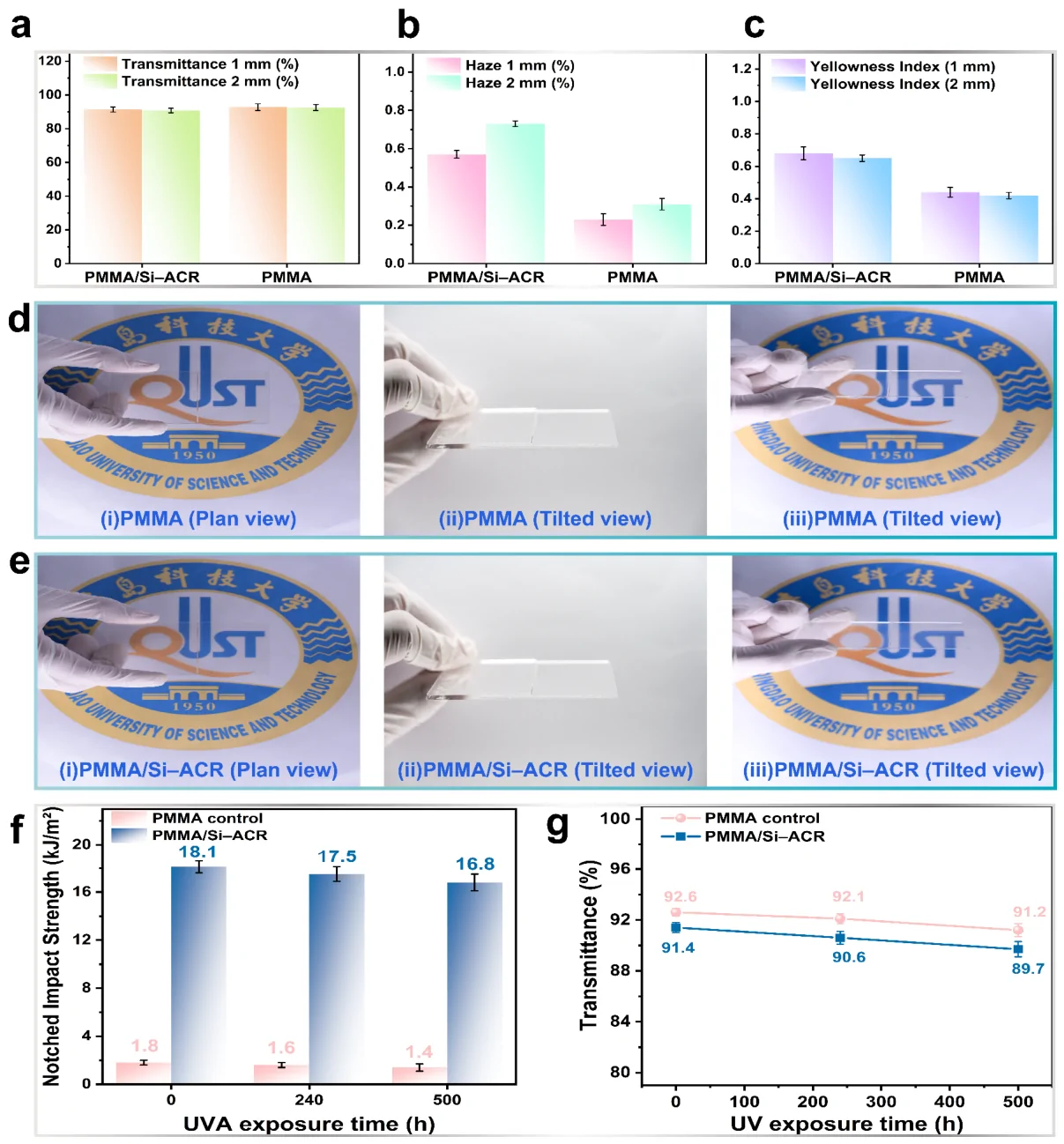
Optical properties and accelerated UVA-aging performance of the PMMA control and PMMA/Si–ACR blend containing 15 wt% Si–ACR. (**a**) Total luminous transmittance, (**b**) haze, and (**c**) yellowness index of 1 and 2 mm specimens. Photographs of (**d**) the PMMA control and (**e**) the PMMA/Si–ACR blend showing their visual transparency. The Chinese characters in the background emblem in (**d**,**e**) denote “Qingdao University of Science and Technology”; the emblem serves as a visual reference for assessing specimen transparency. Changes in (**f**) notched Izod impact strength and (**g**) total luminous transmittance of 1 mm specimens during accelerated UVA exposure for up to 500 h. Error bars represent standard deviations from at least five replicate measurements (*n* ≥ 5).

The retention of mechanical and optical properties during accelerated UVA exposure is shown in Figure 5f,g. After 500 h of exposure, the notched Izod impact strength of the 15 wt% Si–ACR blend decreased from 18.1 to 16.8 kJ/m^2^, corresponding to a retention of approximately 92.8%. In comparison, the PMMA control decreased from 1.8 to 1.4 kJ/m^2^, corresponding to a retention of approximately 77.8%. The total luminous transmittance of the 1 mm modified specimen decreased from 91.4% to 89.7%. These results demonstrate substantial retention of mechanical and optical properties under the specified accelerated-aging conditions. Nevertheless, the different initial impact strengths should be considered when comparing retention percentages. Furthermore, both the PMMA control and modified blends contained 0.5 wt% antioxidant 1076 (Table S3), preventing the individual contributions of the modifier and stabilizer from being independently resolved. UV exposure can induce chemical changes in PMMA, including chain scission and alterations of ester-related groups [37,38]. Because comparative FTIR spectra before and after UVA exposure were not obtained, changes in characteristic chemical bands and specific photostabilization mechanisms cannot be established. Therefore, the present results are interpreted as evidence of macroscopic property retention rather than direct confirmation of suppressed photochemical degradation or long-term outdoor durability.

### 3.4. Mechanistic Interpretation of Toughening and Optical Transparency in PMMA/Si–ACR Blends

**Figure 6:**
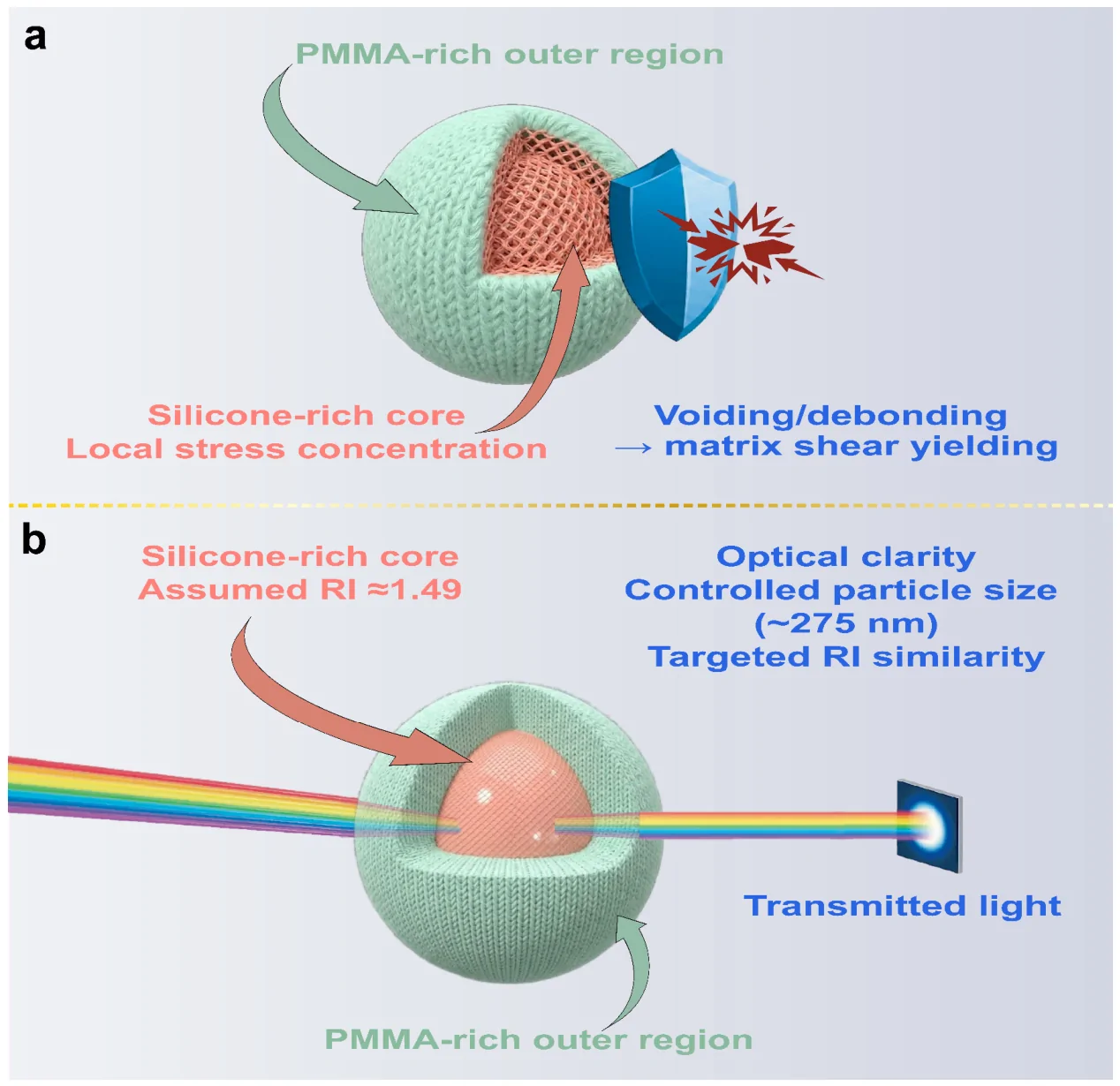
Schematic interpretation of the mechanical and optical behavior of PMMA/Si–ACR blends. (**a**) Proposed stress-concentration/voiding/matrix-shear-yielding sequence, in which local debonding, void formation, or a combination of both can reduce hydrostatic constraint and promote plastic deformation of the surrounding PMMA matrix. (**b**) Optical-design principle based on controlled particle dimensions, a PMMA-rich outer region, and an intended reduction in refractive-index contrast between the phenyl-containing silicone-rich phase and PMMA. In both panels, green and orange–pink represent the PMMA-rich outer region and silicone-rich core, respectively; the colored arrows and symbols in (**a**) illustrate the proposed mechanical response, while the rainbow-colored rays in (**b**) depict light propagation.

Based on the mechanical response and impact-fracture morphology, a plausible toughening mechanism involving local stress concentrations, possible debonding or void formation, and subsequent matrix shear yielding is proposed for the Si–ACR-modified PMMA (Figure 6a). Under impact loading, the compliant silicone-rich cores act as local stress-concentrating domains. Local debonding and/or void formation around the dispersed particles can reduce the triaxial constraint in the surrounding glassy matrix, allowing PMMA to undergo greater plastic shear deformation before unstable crack propagation. This interpretation is consistent with the rough fracture surfaces, plastic ridges, localized cavity-like features, and apparent particle-pull-out sites observed by SEM. The efficiency of this process depends on particle dimensions, interparticle spacing, dispersion, and interfacial characteristics, which explains why an intermediate particle size and modifier content provide the most favorable overall response. Similar cavitation- or debonding-assisted shear-yielding mechanisms have been extensively reported for rubber- and core–shell-toughened glassy polymers, including impact-modified PMMA [39,40,41,42,43,44]. Importantly, the present SEM observations do not distinguish unambiguously among cavitation within the silicone-rich core, separation at the internal core–shell interface, and debonding at the Si–ACR/PMMA matrix interface; therefore, the proposed mechanism should be regarded as consistent with the fracture evidence rather than as direct identification of an individual void-initiation pathway. Further TEM examination of ultrathin sections taken from undeformed and deformed PMMA/Si–ACR blends would be valuable for assessing particle dispersion within the matrix and distinguishing internal particle cavitation from interfacial separation.

The retention of optical transparency is consistent with the combined effects of controlled particle dimensions, dispersion, and the PMMA-rich outer region (Figure 6b). Although the phenyl-containing silicone-rich phase was designed to reduce the refractive-index contrast with PMMA, its actual refractive index was not directly measured. Therefore, the contribution of refractive-index matching remains a plausible design consideration rather than an experimentally verified mechanism [45]. More broadly, differentiated core–shell architectures have been used to balance stiffness and toughness by combining mechanically distinct domains within a single modifier [46]. In the present system, the compliant silicone-rich core promotes mechanical energy dissipation, whereas the PMMA-rich outer region favors compatibility with the surrounding matrix and preservation of optical clarity. Together, these structural features provide a plausible basis for the observed balance between impact resistance and optical transparency. At the selected 15 wt% Si–ACR content, the blend maintained transmittance above 90% and haze below 0.8% while exhibiting substantially improved impact resistance. The observed property retention after accelerated UVA exposure may be associated with the continued contribution of the compliant Si–ACR domains to impact-energy dissipation and the preservation of a favorable mechanical–optical balance during the tested period. However, neither the post-aging particle morphology nor the underlying photochemical degradation pathways were directly characterized. Consequently, the relative contributions of the phenyl-containing silicone-rich phase, the PMMA-rich outer region, and the stabilizer-containing formulation cannot be independently established, and no specific UV-screening or molecular photostabilization mechanism is assigned.

## 4. Conclusions

Silicone–acrylate core–shell particles with a PHSO-derived silicone-rich core and a PMMA-rich outer region were prepared by two-stage seeded emulsion polymerization. Structural and morphological characterization supported the formation of the intended core–shell architecture, while the mechanical and optical results identified an average particle diameter of approximately 275 nm and a Si–ACR content of 15 wt% as providing the most favorable overall property balance. At this composition, the notched Izod impact strength increased approximately tenfold relative to the PMMA control, while transmittance remained above 90% and haze below 0.8%. Fracture-surface observations were consistent with local debonding, void formation, or a combination of both, accompanied by enhanced plastic deformation of the surrounding PMMA matrix. The combination of a compliant silicone-rich core, a PMMA-rich outer region, and controlled particle dimensions therefore offers a promising approach to balancing impact toughness and optical transparency, although the specific contribution of refractive-index matching remains to be verified. The modified PMMA also retained 92.8% of its initial impact strength and a transmittance of 89.7% for the 1 mm specimen after 500 h of accelerated UVA exposure. Overall, this work provides a structure–property design strategy for transparent, impact-resistant PMMA intended for optical and protective components requiring simultaneous toughness, optical clarity, and performance retention under UVA exposure.

## Data Availability

The original contributions presented in this study are included in the article/supplementary material. Further inquiries can be directed to the corresponding authors.

## Supplementary Materials

The following supporting information can be downloaded at: https://www.mdpi.com/article/10.3390/polym18202564/s1, Text S1: Estimation of Volume Fractions and Effective Refractive Index; Figure S1: Effects of Si–ACR particle size and content on the mechanical properties of PMMA/Si–ACR blends; Figure S2: Effects of Si–ACR particle size and content on the optical properties of PMMA/Si–ACR blends; Figure S3: Representative tensile engineering stress–strain curves of PMMA/Si–ACR blends obtained at a crosshead speed of 50 mm/min; Table S1: Parameters used for the optical-design estimation; Table S2: Formulations and reaction parameters for the preparation of Si–ACR particles with different average hydrodynamic diameters; Table S3: Formulations of PMMA/Si–ACR blends and the PMMA control (wt%); Table S4: Effect of Si–ACR particle size on the properties of PMMA/Si–ACR blends at a fixed Si–ACR content of 15 wt%; Table S5: Effect of Si–ACR content on the properties of PMMA/Si–ACR blends using particles with an average diameter of approximately 275 nm; Table S6: Dynamic light scattering characteristics of Si–ACR latexes with different average hydrodynamic diameters.
