# Supplementary material for: Phenyl-Containing Silicone–Acrylate Core–Shell Particles for Transparent Toughening of PMMA with Enhanced Property Retention Under UVA Aging
Source: Polymers (Basel). 2026 Oct 17;18(20):2564. doi: 10.3390/polym18202564 (PMC13709289; doi:10.3390/polym18202564)
Supplement: Supplementary file 1 [file polymers-18-02564-s001.zip › polymers-4610846-supplementary.pdf]

Supplementary Materials for

Phenyl-Containing Silicone–Acrylate Core–Shell Particles for Transparent Toughening of PMMA with Enhanced Property Retention under UVA Aging

Yuao Li[1], Fan Yang[2], Xiang Liu[1], Runxiong Lin[1*], Tao Zhuang[1*]

[1]School of Polymer Science and Engineering, Qingdao University of Science and Technology, Qingdao 266042, P. R. China

[2]School of Materials Science and Engineering, Ocean University of China, Qingdao 266404, P. R. China

Correspondence: lrx@qust.edu.cn (R.L.); zhuangtao@qust.edu.cn (T.Z.)

Contents:

Text S1. Estimation of Volume Fractions and Effective Refractive Index.

Figure S1. Effects of Si–ACR particle size and content on the mechanical properties of PMMA/Si–ACR blends.

Figure S2. Effects of Si–ACR particle size and content on the optical properties of PMMA/Si–ACR blends.

Figure S3. Representative tensile engineering stress–strain curves of PMMA/Si–ACR blends obtained at a crosshead speed of 50 mm/min.

Table S1. Parameters used for the optical-design estimation.

Table S2. Formulations and reaction parameters for the preparation of Si–ACR particles with different average hydrodynamic diameters.

Table S3. Formulations of PMMA/Si–ACR blends and the PMMA control (wt%).

Table S4. Effect of Si–ACR particle size on the properties of PMMA/Si–ACR blends at a fixed Si–ACR content of 15 wt%.

Table S5. Effect of Si–ACR content on the properties of PMMA/Si–ACR blends using particles with an average diameter of approximately 275 nm.

Table S6. Dynamic light scattering characteristics of Si–ACR latexes with different average hydrodynamic diameters.

Text S1. Estimation of Volume Fractions and Effective Refractive Index.

For optical-design estimation, the mass fractions (w_i) of the constituent phases were converted to volume fractions (ϕ_i) using their respective densities (ρ_i) according to eq. S1:

$$\phi_i = \frac{w_i/\rho_i}{\sum_j (w_j/\rho_j)} \tag{S1}$$

The effective refractive index of the Si–ACR particle was then estimated using a linear volume-fraction mixing approximation:

$$n_{\text{eff}} = \sum_i \phi_i \, n_i \tag{S2}$$

where n_i represents the refractive index of each constituent phase. The densities used for the calculation were approximately 1.05 g/cm^3 for the PHSO-derived silicone-rich phase and 1.19 g/cm^3 for PMMA.

For the selected Si–ACR formulation, the nominal PHSO-to-MMA feed mass ratio was 60:40. For the simplified optical-design estimation, this feed ratio was used as an approximation of the mass ratio between the silicone-rich core and the PMMA-rich outer region. Using densities of approximately 1.05 g/cm^3 for the silicone-rich phase and 1.19 g/cm^3 for PMMA, the corresponding estimated volume fractions were approximately 0.630 and 0.370, respectively. Using the approximate refractive-index values listed in Table S1, eq. S2 yields an estimated effective refractive index of approximately 1.49. However, this result follows from the assumed refractive indices of the constituent phases and does not provide independent verification of refractive-index matching. The refractive index of the polymerized silicone-rich phase was not directly measured, and the nominal feed ratio may not exactly represent the final phase composition. Therefore, this simplified calculation serves only as an optical-design estimate rather than experimental validation of the particle refractive index or an optimum phase ratio. The optical response of the PMMA/Si–

ACR blends also depends on particle dimensions, dispersion, morphology, and interfacial structure.

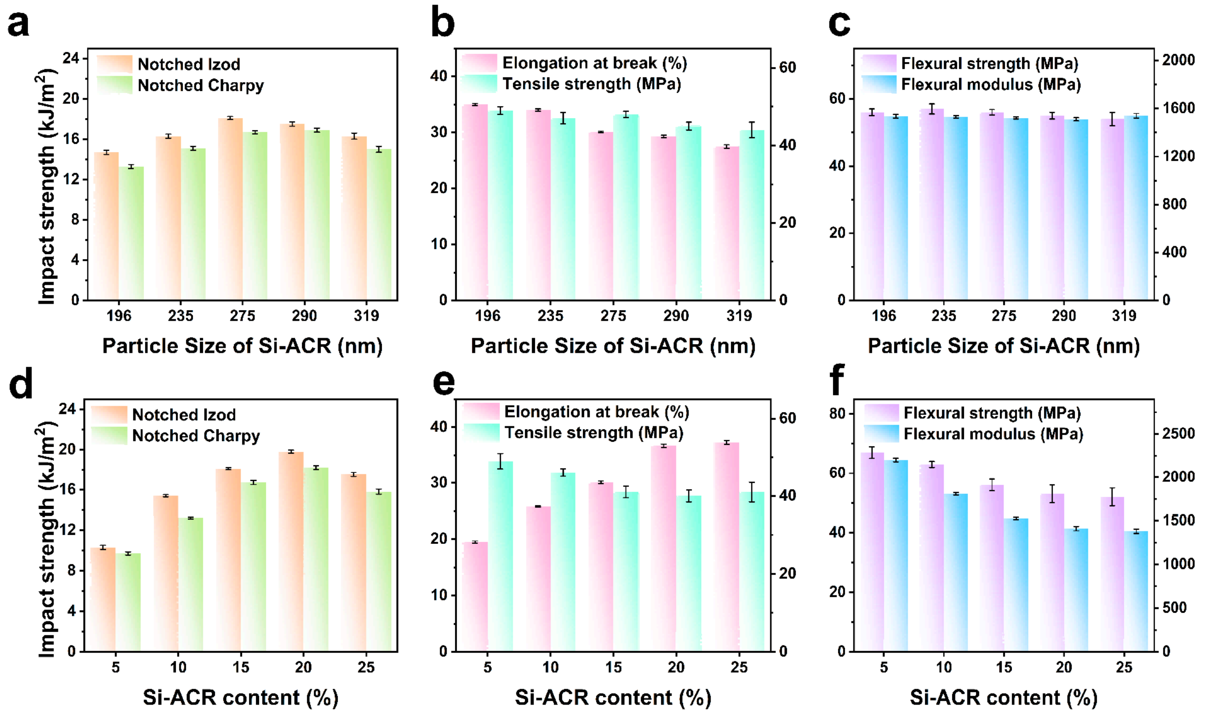

Figure S1. Effects of Si–ACR particle size and content on the mechanical properties of PMMA/Si–ACR blends. (a–c) Particle-size dependence at a fixed Si–ACR content of 15 wt%: (a) notched Izod and Charpy impact strengths, (b) elongation at break and tensile strength, and (c) flexural strength and flexural modulus. (d–f) Si–ACR-content dependence using particles with an average diameter of approximately 275 nm: (d) impact strengths, (e) elongation at break and tensile strength, and (f) flexural strength and flexural modulus. Error bars represent standard deviations from at least five replicate measurements ($n \geq 5$).

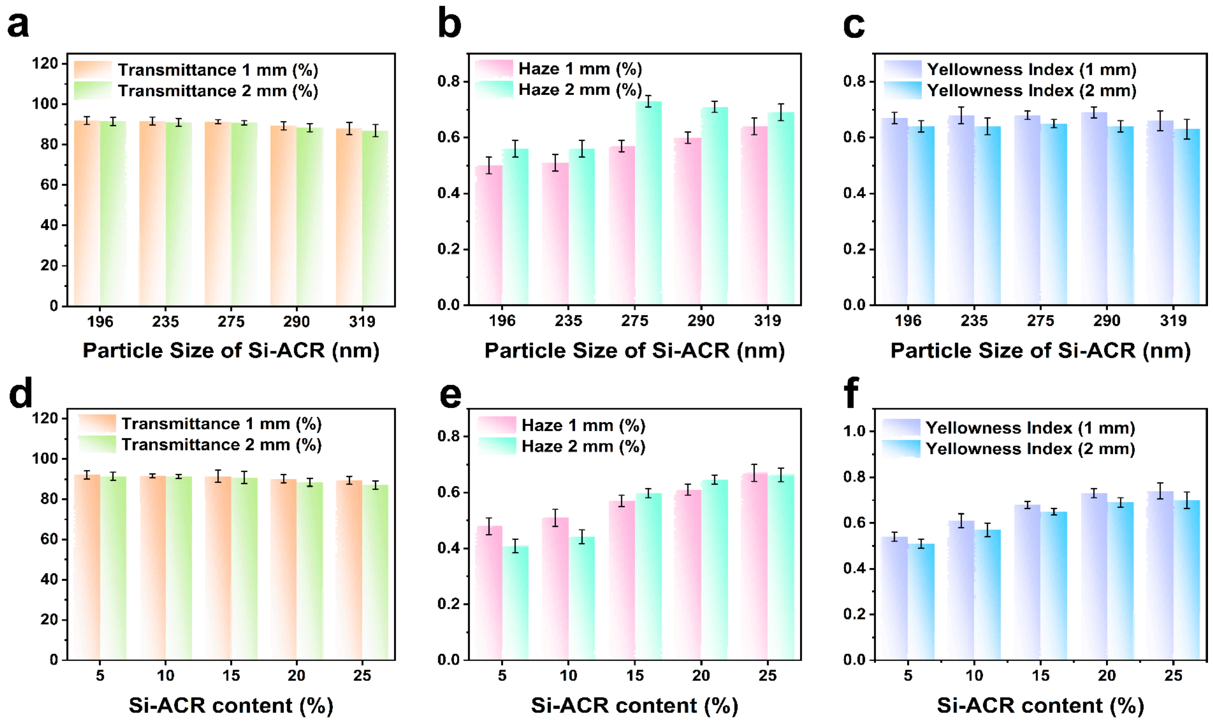

Figure S2. Effects of Si–ACR particle size and content on the optical properties of PMMA/Si–ACR blends. (a–c) Particle-size dependence at a fixed Si–ACR content of 15 wt%: (a) total luminous transmittance, (b) haze, and (c) yellowness index. (d–f) Si–ACR-content dependence using particles with an average diameter of approximately 275 nm: (d) total luminous transmittance, (e) haze, and (f) yellowness index. Optical measurements were performed using 1 and 2 mm specimens. Error bars represent standard deviations from at least five replicate measurements ($n \geq 5$).

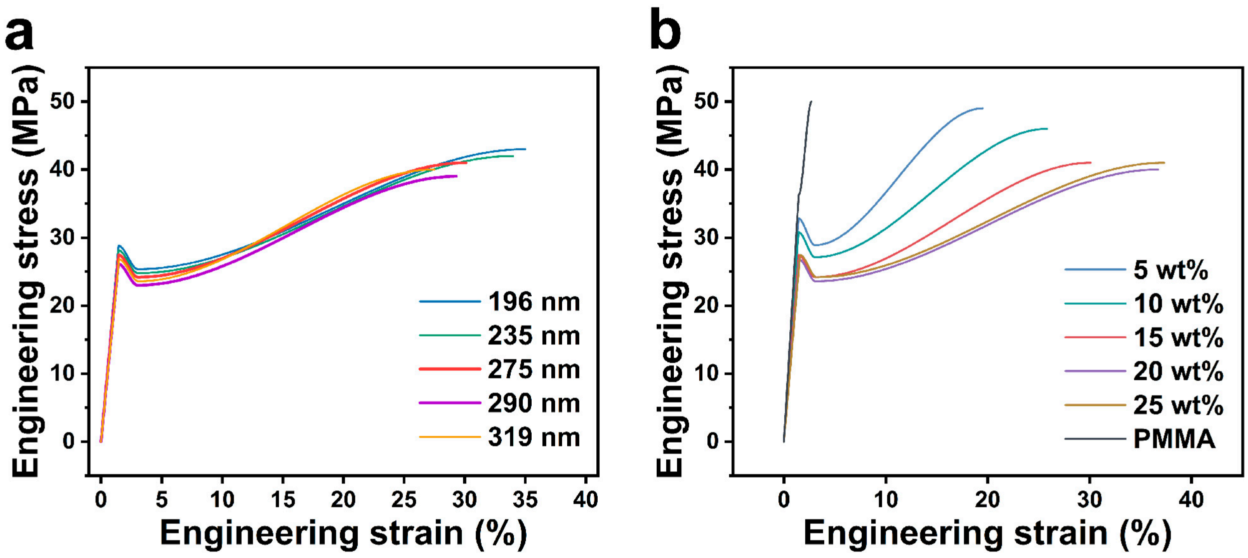

Figure S3. Representative tensile engineering stress–strain curves of PMMA/Si–ACR blends obtained at a crosshead speed of 50 mm/min. (a) Effect of Si–ACR particle size (196, 235, 275, 290, and 319 nm) at a fixed modifier content of 15 wt%. (b) Effect of Si–ACR content (5, 10, 15, 20, and 25 wt%) at a fixed particle diameter of approximately 275 nm, including the PMMA control. Tensile elastic moduli were determined separately at a crosshead speed of 1 mm/min and are summarized in Tables S4 and S5.

Table S1. Parameters used for the optical-design estimation.

Phase	Density (g/cm^3)	Refractive index	Basis
PHSO-derived silicone-rich phase	≈1.05	≈1.49[a]	Estimated
PMMA	1.19	≈1.49[b]	Literature/supplier value

[a] Approximate refractive-index value assumed for optical-design estimation. The refractive index of the PHSO-derived silicone-rich phase after polymerization was not directly measured and should not be regarded as experimental confirmation of refractive-index matching.

[b] Representative literature value for PMMA; the exact value depends on wavelength and measurement conditions.

Table S2. Formulations and reaction parameters for the preparation of Si–ACR particles with different average hydrodynamic diameters.

Component	196 nm	235 nm	275 nm	290 nm	319 nm
Silicone-rich seed stage					
PHSO (g)	60.0	60.0	60.0	60.0	60.0
DVB (g)	0.60	0.60	0.60	0.60	0.60
OP-10 (g)	0.90	0.72	0.60	0.54	0.48
LATEMUL ASK (28 wt% active matter) (g)	2.70	2.16	1.80	1.62	1.44
$NaHCO_3$ (g)	0.30	0.30	0.30	0.30	0.30
Deionized water (g)	120.0	120.0	120.0	120.0	120.0
KPS (g)	0.30	0.30	0.30	0.30	0.30
PMMA-rich outer-region stage					
MMA (g)	40.0	40.0	40.0	40.0	40.0
OP-10 (g)	0.60	0.48	0.40	0.36	0.32
LATEMUL ASK (28 wt% active matter) (g)	1.80	1.44	1.20	1.08	0.96
$NaHCO_3$ (g)	0.20	0.20	0.20	0.20	0.20
Deionized water (g)	60.0	60.0	60.0	60.0	60.0
KPS (g)	0.20	0.20	0.20	0.20	0.20

[a] The column headings represent the average hydrodynamic diameters of the resulting Si–ACR particles determined by dynamic light scattering (DLS).

[b] The listed amounts of LATEMUL ASK refer to the as-received aqueous product containing 28 wt% active matter.

[c] The particle size was regulated by varying the dosages of OP-10 and LATEMUL ASK while maintaining their relative proportion, the nominal PHSO-to-MMA feed mass ratio, and the other polymerization conditions unchanged.

[d] KPS was dissolved in deionized water before addition; the listed values refer to the mass of KPS.

[e] Seed polymerization was conducted at 75 °C under nitrogen for 30 min after KPS addition. The MMA pre-emulsion and the second KPS solution were fed into the seed latex at 75 °C over 120 min, followed by aging at 75 °C for 60 min.

[f] The nominal PHSO-to-MMA feed mass ratio was 60:40 for all formulations. For the simplified optical-design estimation in Text S1, this feed ratio was used as an approximation of the silicone-rich-core/PMMA-rich-outer-region mass ratio and should not be interpreted as a directly measured phase-composition ratio or a uniquely optimized refractive-index-matching composition.

Table S3. Formulations of PMMA/Si–ACR blends and the PMMA control (wt%).

Component	PMMA/Si–ACR-5	PMMA/Si–ACR-10	PMMA/Si–ACR-15	PMMA/Si–ACR-20	PMMA/Si–ACR-25	PMMA control
PMMA	94	89	84	79	74	99
Si–ACR	5	10	15	20	25	—
EBS	0.5	0.5	0.5	0.5	0.5	0.5
Antioxidant 1076	0.5	0.5	0.5	0.5	0.5	0.5

Table S4. Effect of Si–ACR particle size on the properties of PMMA/Si–ACR blends at a fixed Si–ACR content of 15 wt%.

Property	Unit	196 nm	235 nm	275 nm	290 nm	319 nm
Melt flow rate (MFR)	g/10 min	69	63	55	51	45
Notched Izod impact strength	kJ/m^2	14.7	16.3	18.1	17.5	16.3
Notched Charpy impact strength	kJ/m^2	13.3	15.1	16.7	16.9	15.0
Elongation at break	%	35	34	30.1	29.3	27.5
Tensile strength	MPa	43	42	41	39	40
Tensile elastic modulus	MPa	2000	1950	1900	1830	1800
Flexural strength	MPa	56	57	56	55	54
Flexural modulus	MPa	1535	1530	1526	1510	1540
Total luminous transmittance (1 mm)	%	91.9	91.7	91.4	89.3	88.0
Total luminous transmittance (2 mm)	%	91.5	91.0	90.8	88.4	86.9
Haze (1 mm)	%	0.50	0.51	0.57	0.60	0.64
Haze (2 mm)	%	0.56	0.56	0.73	0.71	0.69
Yellowness index (1 mm)	—	0.67	0.68	0.68	0.69	0.66
Yellowness index (2 mm)	—	0.64	0.64	0.65	0.64	0.63

[a] MFR was measured at 230 °C under a load of 10 kg.

[b] Notched Izod and Charpy impact tests were performed using 2.75 and 2 J pendulums according to ISO 180:2023 and ISO 179-1:2026, respectively.

[c] Flexural testing was performed at a crosshead speed of 2 mm/min according to ISO 178:2019.

[d] Tensile strength and elongation at break were determined at a crosshead speed of 50 mm/min according to ISO 527-2:2025. Tensile elastic modulus was determined separately at 1 mm/min using an extensometer, with the modulus calculated over the strain interval of 0.05–0.25% according to ISO 527-1:2019.

Table S5. Effect of Si–ACR content on the properties of PMMA/Si–ACR blends using particles with an average diameter of approximately 275 nm.

Property	Unit	5 wt%	10 wt%	15 wt%	20 wt%	25 wt%	PMMA control
Melt flow rate (MFR)	g/10 min	69	62	55	49	44	81
Notched Izod impact strength	kJ/m^2	10.3	15.4	18.1	19.8	17.5	1.8
Notched Charpy impact strength	kJ/m^2	9.7	13.2	16.7	18.2	15.8	1.2
Elongation at break	%	19.5	25.8	30.1	36.7	37.3	2.7
Tensile strength	MPa	49	46	41	40	41	50
Tensile elastic modulus	MPa	2300	2150	1900	1800	1730	2500
Flexural strength	MPa	67	63	56	53	52	70
Flexural modulus	MPa	2200	1810	1526	1410	1380	2422
Total luminous transmittance (1 mm)	%	92.1	91.6	91.4	90.2	89.4	92.6
Total luminous transmittance (2 mm)	%	91.4	91.3	90.8	88.4	87.1	92.8
Haze (1 mm)	%	0.48	0.51	0.57	0.61	0.67	0.23
Haze (2 mm)	%	0.50	0.54	0.73	0.79	0.81	0.31
Yellowness index (1 mm)	—	0.54	0.61	0.68	0.73	0.74	0.45
Yellowness index (2 mm)	—	0.51	0.57	0.65	0.69	0.70	0.42

[a] MFR was measured at 230 °C under a load of 10 kg.

[b] Notched Izod and Charpy impact tests were performed using 2.75 and 2 J pendulums according to ISO 180:2023 and ISO 179-1:2026, respectively.

[c] Flexural testing was performed at a crosshead speed of 2 mm/min according to ISO 178:2019.

[d] Tensile strength and elongation at break were determined at a crosshead speed of 50 mm/min according to ISO 527-2:2025. Tensile elastic modulus was determined separately at 1 mm/min using an extensometer, with the modulus calculated over the strain interval of 0.05–0.25% according to ISO 527-1:2019.

Table S6. Dynamic light scattering characteristics of Si–ACR latexes with different average hydrodynamic diameters.

Particle-size series (nm)	Z-average ± SD (nm)	PDI ± SD	Intensity peak (nm)	Peak width (nm)
196	196 ± 4	0.142 ± 0.012	202	70
235	235 ± 5	0.125 ± 0.010	242	84
275	275 ± 6	0.118 ± 0.011	281	94
290	290 ± 7	0.136 ± 0.013	299	107
319	319 ± 8	0.161 ± 0.015	329	130

Note: Particle sizes were determined by dynamic light scattering (DLS) at 25 °C. For each formulation, three independently diluted aliquots were prepared from the same latex batch, with three consecutive measurements performed for each aliquot. The Z-average hydrodynamic diameter and polydispersity index (PDI) were obtained from cumulants analysis, whereas the intensity-weighted peak diameter and peak width were determined by particle-size distribution analysis. Values of Z-average and PDI are expressed as mean ± standard deviation (SD) based on three independently prepared aliquots (n = 3), after averaging the replicate measurements for each aliquot.
